# Psilocibin: Current Evidence, Safety Signals, and Challenges in Assessing Potential Multi-Organ Effects

**DOI:** 10.3390/biomedicines14071516

**Published:** 2026-07-06

**Authors:** Kasper Buczma, Katarzyna Kamińska, Kaja Kasarełło, Dagmara Mirowska-Guzel, Dariusz Andrzejuk, Anna Kaczmarek, Agnieszka Cudnoch-Jędrzejewska

**Affiliations:** 1 Laboratory of Centre for Preclinical Research, Chair and Department of Physiology and Pathophysiology, Medical University of Warsaw, Banacha 1b, 02-097 Warsaw, Poland; kasper.buczma@wum.edu.pl (K.B.); kaja.kasarello@wum.edu.pl (K.K.); d.andrzejuk01@gmail.com (D.A.); akr.kaczmarek@gmail.com (A.K.); agnieszka.cudnoch-jedrzejewska@wum.edu.pl (A.C.-J.); 2 Laboratory of Centre for Preclinical Research, Chair and Department of Experimental and Clinical Pharmacology, Medical University of Warsaw, Banacha 1b, 02-097 Warsaw, Poland; dagmara.mirowska-guzel@wum.edu.pl

**Keywords:** cardiotoxicity, psychedelics, PSY, serotonin

## Abstract

**Background/Objectives:** Psilocibin (PSY), a serotonergic hallucinogen, has attracted increasing scientific interest due to its therapeutic potential, particularly in treatment-resistant depression. In parallel with its growing clinical and research relevance, important questions have emerged regarding its safety profile, including potential effects on the liver, kidneys, cardiovascular system, and immune function. The aim of this narrative review was to systematically collect, critically appraise, and organize the dispersed evidence regarding potential multi-organ safety signals associated with PSY exposure. **Methods:** A narrative review was conducted including preclinical studies, pharmacological investigations, available clinical data, and published case reports, including reports of mushroom-related intoxications involving PSY-containing species. All available sources addressing potential toxicological outcomes associated with PSY were considered, regardless of exposure context, in order to reflect the current state of evidence. **Results:** The available evidence base is limited and heterogeneous, consisting primarily of case reports, observational data, and mechanistic preclinical studies. Reported adverse events are rare and frequently confounded by polysubstance use, uncertainty of dose, co-ingestion of other compounds, and lack of exposure standardization. Despite these limitations, biologically plausible mechanisms related to serotonergic receptor activation provide a rationale for further investigation of potential organ-specific effects. However, current controlled clinical data do not provide consistent evidence supporting intrinsic multi-organ toxicity of PSY. **Conclusions:** Current evidence does not confirm clinically meaningful intrinsic multi-organ toxicity of PSY under controlled conditions. Nevertheless, the available literature suggests the presence of potential safety signals that warrant further systematic evaluation. In the context of growing clinical interest in PSY, this review provides a structured synthesis of current knowledge and highlights critical gaps in understanding its organ-specific safety profile.

## 1. Introduction

Despite the tremendous development of medicine, there are still many disease entities that require the search for better and more effective therapeutic solutions. One such example is depression. Major depressive disorder (MDD) affects a substantial portion of the global adult population. A recent cross-national study [1] reported an average lifetime prevalence of 7.5% in males and 13.6% in females, with projected cumulative risk by age 75 of 20.1% for men and 34.0% for women. In the United States, recall-corrected estimates indicate that approximately 17.4% of men and 30.1% of women have experienced at least one major depressive episode during their lifetime [2]. Although initial antidepressant (AD) therapy significantly reduces symptoms of depression in many patients, only 50–60% of persons suffering from MDD respond to the treatment. Moreover, 30–40% of MDD patients never achieve symptom resolution by means of a standard AD therapy [3,4]. Thus, there is a continuous need for the development of new, effective, and safer antidepressant therapies.

Recent research has proposed a potential role of psilocibin (PSY), a naturally occurring psychedelic drug compound produced by more than 200 species of fungi. PSY is primarily a pro-drug that is dephosphorylated by alkaline phosphatase to its active metabolite, psilocin. Psilocin (4-hydroxy-N,N-dimethyltryptamine) is a hallucinogen that acts as an agonist of serotonin receptors 5-HT1A, 5-HT2A, 5-HT2C, and 5-HT4 [5]. PSY and psilocin are listed as Schedule I drugs under the United Nations 1971 Convention on Psychotropic Substances. Hibicke et al. demonstrated that PSY induces rapid antidepressant and anxiolytic effects in vivo, using Wistar Kyoto (WKY) rats—a well-established model for studying mood-related disorders [6]. Similarly, several clinical studies have shown that one or two doses of PSY administered during psychotherapy sessions, under tightly controlled conditions, produce fast-acting and long-lasting antidepressant and anti-anxiety effects in patients with various depressive disorders [7,8,9]. Moreover, in 2017, researchers from Imperial College London conducted two clinical trials investigating the effects of PSY in individuals with treatment-resistant depression. They reported significant clinical improvements, which were accompanied by reduced blood flow in the amygdala, as measured by functional magnetic resonance Imaging (fMRI). Based on patients’ subjective reports, the authors proposed that PSY may effectively ‘reset’ neural circuits associated with depression [10].

The serotonergic pharmacological profile of PSY, particularly its potent agonism at 5-HT2A receptors, may underlie not only its therapeutic effects, but also its potential toxicities. Although PSY is generally considered safe at controlled doses, its effects on various organ systems—especially under conditions of chronic use, high doses or in susceptible populations—remain poorly understood. Existing data on multi-organ toxicity are limited, and systematic studies on potential adverse effects on the cardiovascular, hepatic, renal, and neuroendocrine systems are lacking. This gap in toxicological knowledge underscores the need for comprehensive preclinical and clinical studies to fully characterize the safety profile of PSY, particularly in the context of its novel therapeutic applications.

Since PSY is not currently an approved medicinal product in most jurisdictions, the availability of systematic clinical data regarding its acute and chronic multi-organ safety profile remains limited. In particular, there is a lack of well-controlled long-term studies directly assessing potential organ-specific effects, including the renal, hepatic, cardiovascular, and immune systems, under conditions of repeated exposure or defined dosing regimens. Consequently, the current evidence base is highly heterogeneous and derives from a combination of preclinical pharmacological studies, mechanistic investigations, limited clinical research, and case reports associated with PSY exposure, most commonly in the context of ingestion of PSY-containing mushrooms.

An additional limitation in interpreting the available data is the difficulty in clearly distinguishing between controlled therapeutic exposure and uncontrolled recreational use, as well as the frequent absence of precise dose characterization. These factors substantially limit the ability to establish reliable dose–response relationships and to define potential toxicity thresholds.

Despite these limitations, this review aims to systematically collect and critically integrate the currently available evidence on potential multi-organ safety signals associated with PSY, drawing on clinical data, case reports, and mechanistic preclinical literature in order to provide a comprehensive and contextualized overview of the current state of knowledge.

## 2. Materials and Methods

The present study was designed as a narrative review aimed at summarizing and critically evaluating the available evidence regarding the potential toxicity of PSY and its effects on selected organ systems, with particular emphasis on serotonergic mechanisms. The literature search was conducted between January and May 2026 and included studies published up to May 2026.

Relevant articles were identified using major biomedical databases, including PubMed, Scopus, Web of Science, and Google Scholar. The search strategy employed combinations of keywords and Boolean operators (AND/OR), including “PSY toxicity”, “psilocin toxicity”, “PSY AND cardiovascular effects”, “PSY AND liver effects”, “PSY AND kidney effects”, “PSY AND platelets”, “PSY AND glial cells”, and “serotonergic toxicity AND PSY”.

The selection of literature was not restricted to a formal systematic protocol. Eligible sources comprised original in vitro, in vivo, and clinical studies, as well as review articles addressing the pharmacology, toxicology, and organ-specific effects of PSY and psilocin. Non-relevant publications and purely anecdotal reports were excluded based on content screening. The selection process involved screening titles, abstracts, and full texts, with duplicate records removed where applicable.

Data extraction focused on reported toxic effects and physiological impacts on the cardiovascular system, kidneys, liver, glial cells, and platelets, with particular attention to mechanisms involving serotonin receptors, especially 5-HT2A.

As the present work was conducted as a narrative review rather than a systematic review, PRISMA guidelines were not formally applied. Studies were selected based on their relevance to the objectives of the review and their contribution to understanding the potential organ-specific effects of PSY and psilocin.

## 3. Findings

### 3.1. PSY: Potential Therapy and Mechanism of Action

As of 2022, over 60 clinical trials investigating the therapeutic effects of PSY have been registered with the United States National Institutes of Health. While short-term effects have been well documented, the long-term efficacy and safety of PSY therapy remain to be determined, as most trials are still ongoing. However, preliminary results suggest that PSY therapy is effective in treating depression, smoking cessation, alcohol use disorder, and obsessive–compulsive disorder [11].

Earlier PSY trials typically dosed according to body weight, with doses generally ranging from 0.2 to 0.4 mg/kg per session. Protocols involved either a single dose or two doses separated by an average washout period of 3 to 4 weeks. More recent clinical trial protocols have shifted toward using a fixed PSY dose—most commonly 25 mg—which aligns with the approximate 0.3 mg/kg weight-based dosing used previously [12]. This fixed 25 mg dose approach has been validated in a recent secondary analysis of prior trial data, which found no significant differences in psychedelic effects when compared to weight-based doses of 0.29 mg/kg and 0.43 mg/kg [13].

PSY is rapidly dephosphorylated in the body to psilocin, its active metabolite, which acts as an agonist of several serotonin receptors with highest affinity for 5-HT2A receptors—key mediators of its psychedelic effects. It also exhibits lower affinity binding to 5-HT1 receptors, including the 5-HT1A and 5-HT1D subtypes. Additionally, the effects of psilocin are modulated through interactions with 5-HT2C receptors [14].

Notably, the psychotomimetic (psychosis-like) effects induced by psilocin can be blocked in a dose-dependent manner by ketanserin, a selective antagonist of the 5-HT2A receptor. This finding underscores the critical role of 5-HT2A receptor activation in the manifestation of psychedelic effects. However, multiple lines of evidence indicate that psilocin’s interactions with other non-5-HT2 receptors also contribute significantly to its subjective and behavioral effects. These additional receptor interactions highlight the complexity of psilocin’s mechanism of action and suggest that its psychoactive profile involves a broader serotonergic modulation beyond the 5-HT2A receptor alone [15,16].

PSY use is not free from adverse effects. Common responses include pupil dilation (93%); changes in heart rate (100%), including increases (56%), decreases (13%), and variable responses (31%); changes in blood pressure (84%), including hypotension (34%), hypertension (28%), and general instability (22%); changes in stretch reflex (86%), including increases (80%) and decreases (6%); nausea (44%); tremor (25%); and dysmetria (16%). The temporary increases in blood pressure caused by the drug can be a risk factor for users with pre-existing hypertension [17]. These qualitative somatic effects caused by PSY have been corroborated by several early clinical studies [17,18,19,20,21]. According to the largest controlled clinical study of PSY to date at King’s College London, volunteers who received doses of PSY experienced some changes in mood and perception but no negative effects on cognitive or emotional functioning. These systemic physiological effects of PSY provide a basis for further discussion of its underlying pharmacological mechanisms, particularly its serotonergic activity within the cardiovascular system.

### 3.2. PSY: 5-HT Effect on the Cardiovascular System

5-HT exerts important effects on the cardiovascular system, with mechanisms that are complex and dependent on receptor subtype, tissue distribution, and local concentration. One of the key effects is vasoconstriction, particularly in small arteries and arterioles. Experimental studies have demonstrated 5-HT-induced vasoconstriction in coronary arteries [22], and reperfusion models suggest that elevated 5-HT levels may contribute to cellular injury, including apoptosis and necrosis [23]. In humans, vasoconstrictive responses to 5-HT have been observed in the presence of functional endothelium [24,25], and partially attenuated by 5-HT2 receptor antagonism [26]. Studies in isolated human pulmonary vessels further indicate that 5-HT-induced vasoconstriction involves both 5-HT1 and 5-HT2 receptors, with receptor expression (including 5-HT4, 5-HT2A and 5-HT1D) confirmed in these tissues [27].

**Figure 1:**
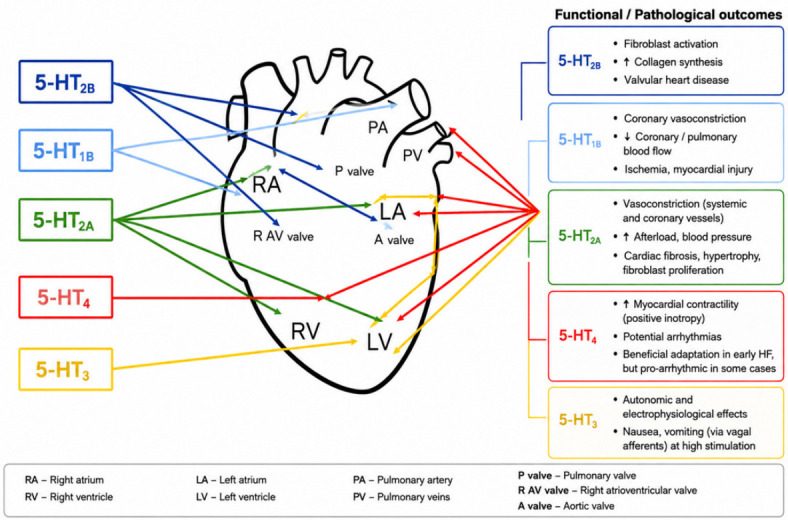
Effects of serotonin (5-HT) receptor subtypes on the cardiovascular system and associated functional and pathological outcomes.

Beyond acute vascular effects, serotonin receptors also play a role in cardiac remodeling (Figure 1). The 5-HT2A receptor, expressed in cardiomyocytes, is upregulated in hypertrophic conditions and left ventricular dysfunction, where its activation has been associated with fibrosis, fibroblast proliferation, and collagen synthesis [28,29,30,31]. Similarly, 5-HT2B receptors expressed on cardiac fibroblasts have been linked to pro-inflammatory and pro-remodeling signaling, including increased levels of IL-6, TNF-α, IL-1β, and norepinephrine in heart failure states [32]. Importantly, excessive 5-HT2B stimulation has been associated with valvular heart disease characterized by valvular thickening and regurgitation.

The 5-HT4 receptor, structurally and functionally linked to Gs protein signaling, is expressed in both atrial and ventricular tissue, with expression increasing in ventricular dysfunction [33,34]. Its activation has been associated with positive inotropic and lusitropic effects similar to β1-adrenergic stimulation, which may be beneficial in heart failure but can also contribute to arrhythmogenic risk under pathological conditions [35]. Increased 5-HT4 expression has been observed in early stages of hypertension-induced hypertrophy, correlating with enhanced serotonergic inotropic responses [5].

In addition to receptor-mediated mechanisms, serotonin may exert receptor-independent intracellular effects. Oxidative metabolism of 5-HT in mitochondria can generate reactive oxygen species, contributing to apoptosis and necrosis [34]. Furthermore, serotonin may covalently modify intracellular and surface proteins via transglutaminase-mediated reactions, potentially altering protein function in various cell types, including cardiomyocytes [36].

Finally, serotonin plays a bidirectional role in blood pressure regulation. Depending on receptor subtype activation, it may induce vasoconstriction via 5-HT2A receptors or vasodilation via 5-HT1-mediated pathways, reflecting its context-dependent vascular effects.

Beyond its cardiovascular effects, PSY also influences serotonergic pathways in other peripheral organs, including the urinary and hepatic systems.

### 3.3. 5-HT Effects on the Urinary and Hepatic Systems

In the urinary system, 5-HT receptors are distributed in various parts, including the glomeruli, renal tubules, and blood vessels. There are several mechanisms by which 5-HT and its receptors influence kidney function. 5-HT affects the smooth muscle tone of blood vessels; binding of 5-HT to 5-HT2A receptors can induce vasoconstriction, reducing renal blood flow. Conversely, activation of 5-HT1 receptors may cause vasodilation, improving blood flow [37].

5-HT also plays a role in the filtration process within the kidneys by modulating glomerular vascular tone, thereby affecting the glomerular filtration rate (GFR), which is essential for kidney function. Moreover, 5-HT influences the secretion of renin, a hormone involved in regulating blood pressure and fluid balance. Renin acts through the renin–angiotensin–aldosterone system to regulate water and electrolyte homeostasis, which directly impacts kidney function [38]. Additionally, 5-HT affects the renal tubules by modulating the transport of ions such as sodium and potassium, as well as water reabsorption—processes that are vital for maintaining electrolyte balance and blood pressure [37].

The effects of 5-HT on the liver are less extensively studied compared to its roles in organs like the heart or kidneys. Nevertheless, 5-HT interacts with the liver in multiple ways. Various studies have identified numerous serotonergic receptors in the epithelium of the gastrointestinal tract, including hepatic tissues [39]. Among these, 5-HT2A receptors are particularly important, serving as primary sites for PSY activity. Stimulation of these receptors can lead to symptoms such as abdominal pain, discomfort, nausea, and vomiting [39].

5-HT also regulates smooth muscle tone in hepatic vasculature. Activation of 5-HT2A receptors causes vasoconstriction, which can alter hepatic blood flow. These changes affect oxygen and nutrient delivery to the liver, as well as the clearance of metabolic waste products [40]. Moreover, 5-HT3 receptors, which are present in various tissues including the liver, modulate inflammatory and metabolic processes. Consequently, serotonin may play a role in regulating inflammatory responses implicated in liver diseases such as steatosis and cirrhosis.

Furthermore, 5-HT influences liver metabolism, including detoxification, protein synthesis, and lipid metabolism. The liver is the primary organ responsible for 5-HT metabolism, as most 5-HT breakdown and inactivation—mediated by enzymes such as monoamine oxidase (MAO)—occur there [41]. In patients with chronic liver diseases like cirrhosis, altered 5-HT levels are often observed. Elevated 5-HT in the bloodstream may be linked to impaired hepatic microcirculation, potentially contributing to portal hypertension (increased pressure in the portal veins) and the progression of liver failure [42].

There is also some evidence suggesting that 5-HT may influence bile secretion, although this area remains underexplored. Additionally, 5-HT’s effect on intestinal motility may indirectly impact the biliary system [41].

In pathological conditions such as renal failure, hypertension, or metabolic diseases, alterations in 5-HT levels and receptor function can exacerbate renal dysfunction. For instance, chronic activation of 5-HT2A receptors has been associated with renal fibrosis [43].

### 3.4. Evidence of PSY Cardiotoxic Effects

Despite growing interest in the medical properties of psilocibin (PSY), data on its potential cardiotoxicity remain limited. Most available evidence originates from case reports of mushroom poisoning and a small number of clinical studies, while animal models exploring underlying molecular mechanisms are scarce.

Reported electrocardiographic abnormalities associated with PSY administration, including ST-segment elevation (Nef et al., 2009 [28]) and QT interval prolongation (Li et al., 2019 [29]), suggest a potential influence on cardiac ion channel activity. Current evidence indicates that its active metabolite, psilocin, rather than PSY itself, is primarily responsible for these electrophysiological effects. In animal studies, psilocin administration in rats induced ECG abnormalities, impaired intraventricular conduction, and cardiac arrhythmias [30].

Further insight was provided by a Phase I clinical trial in 12 healthy participants using concentration–QTc (C-QTc) analysis following single ascending doses of PSY (0.3–0.6 mg/kg). A statistically significant but weak relationship between psilocin plasma concentration and QTc prolongation was observed. At a therapeutically relevant dose of 25 mg, mean maximum psilocin concentration was 18.7 ng/mL, with a mean QTcF prolongation of 2.1 ms (upper 90% CI: 6.6 ms). However, higher doses corresponding to 42–59 mg PSY were predicted to exceed regulatory concern thresholds (Dahmane et al., 2021 [44]; In contrast, another clinical study involving 23 healthy participants reported no significant QTc differences between 25 mg PSY and placebo (Becker et al., 2022 [45]). Overall, these findings suggest that therapeutic doses are associated with limited QTc risk, although higher exposures may carry increased proarrhythmic potential.

From a mechanistic perspective, PSY exerts its primary effects via agonism of 5-HT2A receptors, which underlies both its psychedelic and autonomic cardiovascular effects. Activation of serotonergic pathways may increase sympathetic nervous system activity, resulting in transient, dose-dependent increases in heart rate and blood pressure. These effects are generally short-lasting but may be clinically relevant in individuals with pre-existing cardiovascular disease.

In contrast, PSY shows weak affinity for cardiac 5-HT4 receptors located in the sinoatrial node and atrial tissue, which are involved in heart rate regulation and impulse conduction. Therefore, transient tachycardia observed after PSY administration is considered to result primarily from central autonomic activation rather than direct myocardial 5-HT4 stimulation.

Clinical data support these observations. A meta-analysis by Yerubandi et al. (2024) [46], evaluating PSY in psychiatric indications, reported increased heart rate and blood pressure across multiple studies. In one trial, elevated blood pressure occurred in 76% of participants receiving 21 mg PSY, while another reported systolic BP > 160 mmHg in 34% and diastolic BP > 100 mmHg in 13% following high doses. Mild increases were also observed at lower doses, contributing to variability between studies.

Similarly, a clinical study in 20 healthy participants (aged 22–43 years) with prior psychedelic experience showed dose-dependent but transient increases in blood pressure following PSY administration (10–30 mg/70 kg), with mean values of 138/80, 142/85, and 140/87 mmHg, respectively.

From an ion channel perspective, PSY-induced blockade of hERG channels does not appear to fully explain its cardiac effects, and is unlikely to be the primary mechanism underlying QT prolongation observed in clinical settings (Dahmane et al., 2021 [44]).

Finally, potential long-term cardiovascular safety concerns have focused on 5-HT2B receptors expressed in cardiac valves, whose chronic activation is associated with valvular fibrosis. However, there is currently no evidence that PSY induces sustained 5-HT2B stimulation sufficient to cause structural cardiac remodeling, likely due to its short duration of action and intermittent exposure.

### 3.5. Evidence of PSY Hepatotoxic Effects

In standard doses and under controlled conditions, psilocibin (PSY) does not show clear evidence of direct liver injury. Nevertheless, adverse hepatic reactions may occur in specific situations, particularly in cases of misuse or concomitant use with other hepatotoxic substances. Overall, current data suggest a relatively limited direct impact of PSY on liver function, although several factors may modify its risk profile.

After oral administration, PSY acts as a prodrug and is rapidly metabolized in the liver to its active form, psilocin. This biotransformation occurs mainly via dephosphorylation mediated by non-specific phosphatases rather than direct involvement of cytochrome P450 enzymes. However, some authors suggest a potential indirect role of CYP isoenzymes such as CYP2D6 and CYP3A4 in overall hepatic metabolic variability [12,47]. In individuals with impaired liver function, including those with chronic liver disease or cirrhosis, enzymatic activity may be reduced or altered. This may affect PSY metabolism, leading to delayed clearance and altered exposure to psilocin or its metabolites [48]. Such changes may theoretically increase the risk of serotonergic overstimulation and related adverse effects [49].

Furthermore, impaired hepatic metabolism may influence both the duration and intensity of psilocin’s effects on central and peripheral 5-HT receptors, potentially exacerbating cardiovascular or neurological adverse reactions, including arrhythmias or neurotoxic-like symptoms [14,15]. Therefore, liver function represents an important consideration in therapeutic contexts, where dose adjustments or alternative strategies may be required to minimize potential risks.

On the other hand, preclinical studies suggest potential beneficial metabolic effects of PSY under experimental conditions. In a C57BL/6 mouse model fed a high-fat, high-fructose diet (HFHFD) for 17 weeks, one group received PSY at a dose of 0.05 mg/kg body weight daily, while the control group received vehicle via oral gavage. PSY, without affecting food intake, reduced hepatic steatosis and body weight by 12% and restored plasma and hepatic triglyceride levels in mice fed a standard diet. A significant reduction in fasting glucose and area under the curve (AUC) was observed in the oral glucose tolerance test. PSY was also shown to restore hepatic expression of genes involved in lipid localization and catabolic processes, as well as insulin receptor and IRS-1 protein expression, which were altered in metabolic dysfunction-associated steatotic liver disease (MASLD). These findings suggest that PSY may modulate hepatic metabolic pathways under experimental conditions rather than exerting hepatotoxic effects.

In addition to hepatic involvement, evidence suggests that PSY may also affect renal physiology, raising the possibility of nephrotoxic effects.

### 3.6. Evidence of PSY Nephrotoxic Effects

The first reported case of acute kidney injury (AKI) temporally associated with psilocibin (PSY)-containing mushroom ingestion was described in 1992. A 20-year-old woman was admitted with nausea, vomiting, diarrhea, bloating, and abdominal pain occurring 7 days after consumption of so-called “magic mushrooms.” Clinical evaluation revealed mild proteinuria, hematuria, and hypertension (blood pressure 160/100 mmHg) [49]. Histopathological examination demonstrated mild non-ischemic necrosis of the renal tubular epithelium within the renal cortex, without evidence of glomerular injury. Tubular epithelial damage was proposed to impair nephron function and reduce renal excretory capacity, leading to accumulation of metabolic waste products [50]. Based on clinical presentation, temporal association, and an unremarkable medical history, the episode was attributed to ingestion of PSY-containing mushrooms [51].

A similar case was reported in 2019 by Myron and colleagues in a 15-year-old Canadian male who ingested several *Psilocybe cubensis* mushrooms. The patient presented with nausea and abdominal pain and developed impaired renal function, with creatinine rising from 207 to 444 µmol/L (reference range 65–121 µmol/L) and elevated urea of 13.5 mmol/L (reference range 3.0–7.0 mmol/L). PSY was confirmed in the ingested material using liquid chromatography–mass spectrometry. Urinalysis showed microscopic hematuria (5–10 RBC/HPF) without proteinuria, leukocyturia, or casts. Renal ultrasound revealed bilaterally normal-sized kidneys with increased cortical echogenicity, suggestive of parenchymal involvement [52].

Another case involved a 31-year-old previously healthy female who developed proteinuria and microscopic hematuria following ingestion of PSY-containing mushrooms. Renal biopsy demonstrated features consistent with vascular-predominant acute thrombotic microangiopathy [53].

Current pharmacological and clinical data indicate that PSY and its active metabolite psilocin are unlikely to be intrinsically nephrotoxic. In controlled clinical studies of PSY-assisted therapy, no consistent signal of clinically significant acute kidney injury has been observed. Reported cases of AKI following ingestion of PSY-containing mushrooms are therefore rare and are most plausibly explained by indirect or multifactorial mechanisms, including dehydration secondary to gastrointestinal symptoms, sympathetic activation, hyperthermia, co-ingestion of other substances, or misidentification of mushroom species.

Beyond nephrotoxicity, PSY has also been implicated in the modulation of immune system activity via serotonergic mechanisms.

### 3.7. PSY and Immunity

Multi-organ toxicity can also affect the immune system, both directly and indirectly, depending on the type of toxin and the organs that are affected.

PSY, in addition to its well-documented effects on the central nervous system, may also affect the immune system. Serotonin receptors, to which the active form of PSY, psilocin, binds, are also found on immune cells such as lymphocytes, monocytes and macrophages. Preliminary studies suggest that PSY can modulate the inflammatory response and affect the cytokine profile, indicating its potential immunomodulatory effects [54].

In general, the knowledge regarding the effects of psychedelics on immunity is scant. It is proposed that PSY exerts anti-inflammatory activity as the agonist of serotonergic receptor 5-HT2A [55]. Literature data shows that the 5-HT2A agonist, (R)-2,5-dimethoxy-4-iodoamphetamine [(R)-DOI], results in a decrease in the expression of IL-6 and IL-1b cytokines, Mcp-1 and Cx3cl1 chemokines and Icam-1 and Vcam-1 integrins in the model of TNF-α-induced systemic inflammation in mice. Although the mechanism is not so evident, because the 5-HT2A antagonists also presented anti-inflammatory effects [56]. The next piece of evidence was presented in the mouse asthma model, where [(R)-DOI] administration caused a decrease in IL-6 and Mcp-1 expression, eosinophil infiltration into the lungs, and mucus production [57]. A decrease in the expression of IL-6, Vcam1, and TNF-α was likewise shown in the aortas of mice with high-fat-diet-induced inflammation [55]. In vitro studies performed using the 3D human EpiIntestinal tissue stimulated with TFN-α and IFN-γ to mimic the inflammatory conditions showed that PSY decreases the levels of TNF-α, IFN-γ, IL-6, IL-8, MCP-1 and GM-CSF [58]. In human volunteers, the single dose (0.22 mg/kg) of PSY given orally did not cause significant changes in TNF serum levels 23 h after administration [59].

Given that immune cells express serotonergic receptors, emphasize the possible role of psychedelics and PSY as anti-inflammatory agents [60].

Another way of influencing the immune system is via the hypothalamic–pituitary–adrenal axis. It was presented that the 5-HT2A agonist, (±)-1-(2,5-dimethoxy-4-iodophenyl)-2-aminopropane HCl (DOI), stimulated the corticotropin-releasing factor neurons in the hypothalamus, thus stimulating ACTH release in rats [61]. PSY administration to healthy human volunteers resulted in increased plasma levels of both ACTH and cortisol [62].

An interesting experiment was conducted in healthy volunteers, aiming to analyze the effect of PSY on both immunity and stress levels. Inflammatory markers were significantly decreased either 80 min after the PSY administration (TNF-α) or when measured seven days after PSY administration (IL-6, CRP). Cortisol levels, on the other hand, were elevated just after PSY administration, but not seven days after. Additionally, cortisol levels were elevated after applying the psychosocial stressor, both in the placebo and PSY group.

No clinical trials have examined the effect of PSY on immunity to date, so assessing potential risks is not possible. Based on the literature data, PSY presents a rather safe, anti-inflammatory profile, but further investigation, possibly in the context of contribution to organ toxicity, is needed.

### 3.8. Serotonergic Modulation and Bleeding Risk: Implications for PSY

It is also important to consider bleeding risk in the broader context of serotonergic modulation [63]. Selective serotonin reuptake inhibitors (SSRIs) are a well-established example of drugs associated with an increased risk of bleeding, an effect mechanistically linked to the role of serotonin in platelet function [64]. Platelets do not synthesize serotonin de novo but acquire it from the circulation via the serotonin transporter (SERT); serotonin release during platelet activation contributes to primary hemostasis by amplifying platelet aggregation. Inhibition of this process results in reduced intraplatelet serotonin levels and impaired aggregation, thereby increasing bleeding susceptibility [65].

Although this mechanism has been primarily described in the context of SSRI use, it underscores the central role of serotonin in platelet-mediated hemostasis. PSY, through its distinctly serotonergic pharmacological profile—predominantly acting as an agonist of 5-HT receptors—may theoretically influence hemostatic processes, despite its mechanism of action being fundamentally different from serotonin reuptake inhibition [66]. While direct evidence assessing the impact of PSY on bleeding risk is currently lacking, the established involvement of serotonin in platelet function supports a cautious interpretation and highlights the need for further investigation.

### 3.9. Safety Comparison of PSY-Assisted Therapy and Standard Antidepressant Treatment

From a safety perspective, PSY used in the treatment of depression differs substantially from standard antidepressant therapies in both risk profile and mode of administration. PSY is typically administered in controlled clinical settings as a limited number of sessions, in contrast to conventional antidepressants such as SSRIs, SNRIs, or tricyclic antidepressants, which are used chronically over weeks to years (Carhart-Harris et al., 2016 [7]).

Standard antidepressants are associated with a well-characterized profile of long-term adverse effects, including sexual dysfunction, weight gain, sleep disturbances, discontinuation symptoms, and, in some cases, dose-dependent cardiovascular or hepatic risks depending on the drug class (Stahl, 2013; Shelton, 2003) [67,68].

In terms of acute and long-term safety, PSY is more commonly associated with short-lasting psychological reactions, whereas antidepressants carry a greater burden of persistent physiological side effects. From a cardiovascular perspective, PSY induces transient sympathetic activation with short-term increases in blood pressure and heart rate, while SSRIs are generally cardiovascularly neutral and SNRIs may contribute to sustained increases in blood pressure in some patients (Importantly, although PSY has serotonergic activity, clinically significant serotonin syndrome appears rare under controlled conditions, while potential drug–drug interactions remain an important consideration, particularly when combined with other serotonergic agents (Carhart-Harris et al., 2016 [7])).

Overall, the key difference in safety profiles is not related to confirmed organ toxicity but rather to the duration and nature of exposure: PSY produces acute, time-limited psychopharmacological effects under supervised conditions, whereas standard antidepressants exert continuous neurochemical modulation with a well-established long-term safety profile but a higher incidence of persistent adverse effects.

Organ-specific toxicity of psilocybin: proposed mechanisms, clinical manifestations, and supporting evidence (Table S1).

## 4. Conclusions

Abnormalities in the serotonergic system may play an important role in the pathophysiology of multiple cardiovascular disease states such as systemic hypertension, primary pulmonary hypertension and peripheral vascular disease but also in the toxicity of other organs. In general, 5-HT is not directly associated with toxic effects on the liver or kidneys under normal conditions, but excess or abnormal 5-HT can have potentially harmful effects on the body.

However, PSY appears to have relatively low toxic potential compared to other psychoactive substances. A number of animal and human studies have not observed serious brain damage from its acute use, and it is even suggested that it may have potential therapeutic benefits, such as treating depression, anxiety or addiction. However, when PSY is abused, adverse effects such as cardiovascular disorders can occur. Data also indicates the possibility of acute kidney damage. In addition, when PSY is used in combination with other substances, such as alcohol, painkillers or other substances metabolized by the liver, there may be a synergistic burden on the liver, which may increase the risk of liver damage.

In summary, considering the potential medical applications of PSY, further long-term preclinical and clinical studies are necessary, including both single-dose and chronic dosing regimens, in order to more fully characterize its safety profile. Particular attention should be paid to evaluating the effects of both therapeutic and recreational doses of PSY on organ function and potential long-term adverse effects.

At the same time, assessing the safety of recreational doses remains particularly challenging, as recreational use most commonly involves the consumption of PSY-containing mushrooms rather than administration of a synthetic compound with a precisely defined dose. Moreover, the content of PSY and other biologically active compounds in mushrooms may vary substantially depending on species, cultivation conditions, and preparation methods, making accurate dose estimation and interpretation of toxicological findings difficult.

## 5. Study Limitations

One of the main limitations of this study is the lack of available long-term toxicological data on PSY, as the substance has not yet been approved as a registered medicinal product. Consequently, there are no large-scale human studies evaluating its potential multi-organ toxicity or long-term adverse effects. Another important limitation is the inability to reliably estimate the ingested dose of PSY, since most reported cases involve the consumption of PSY-containing mushrooms rather than standardized synthetic formulations. Approximately 200 mushroom species contain PSY, and variations in species composition, alkaloid concentration, and preparation methods may substantially affect toxicity profiles. Therefore, direct comparisons between therapeutic and recreational exposure are difficult, and the causal relationship between PSY dose and observed organ toxicity remains uncertain.

A further limitation is that evidence regarding potential nephrotoxicity and hepatotoxicity is derived predominantly from isolated case reports associated with mushroom ingestion. Although the mushroom species described in these reports were identified by the respective authors, interpretation of these findings remains challenging due to possible confounding factors, including variability in PSY and other alkaloid concentrations, dehydration, concomitant substance use, co-exposure to additional mushroom-derived compounds, and differences in preparation methods. These factors limit the ability to establish a direct causal relationship between PSY exposure and organ injury.

Another limitation of this review is its narrative character and the methodology used for literature selection. The manuscript was intended as a broad overview of the currently available evidence rather than a formal systematic review. Additionally, the available literature on PSY-related toxicity remains relatively limited and consists largely of case reports, preclinical studies, and mechanistic observations, with comparatively few long-term clinical investigations. Therefore, some conclusions should be interpreted with caution, particularly in the context of assessing potential long-term organ toxicity. Nevertheless, the collected evidence highlights the need for further well-designed experimental and clinical studies to better characterize the safety profile of PSY.

## Data Availability

No new data were created or analyzed in this study.

## Supplementary Materials

The following supporting information can be downloaded at: https://www.mdpi.com/article/10.3390/biomedicines14071516/s1, Table S1. Summary of the available evidence regarding organ-specific toxicity associated with psilocybin. The table presents the proposed biological mechanisms, reported clinical manifestations, and the type of evidence supporting each association across major organ systems. Current evidence indicates that transient cardiovascular effects are the most consistently documented adverse events, whereas evidence for hepatic, renal, immunological, hematological, and overall systemic toxicity remains limited and is derived primarily from preclinical studies, case reports, and mechanistic investigations.

## Abbreviations

The following abbreviation is used in this manuscript:

PSY	Psilocibin

## References

[1] Salari N., Babajani F., Hosseinian-Far A., Hasheminezhad R., Abdoli N., Haydarisharaf P., Mohammadi M. (2024). Global prevalence of major depressive disorder, generalized anxiety, stress, and depression among infertile women: A systematic review and meta-analysis. Arch. Gynecol. Obstet..

[2] Tam J., Mezuk B., Zivin K., Meza R. (2020). U.S. simulation of lifetime major depressive episode prevalence and recall error. Am. J. Prev. Med..

[3] Amsterdam J.D., Fava M., Maislin G., Rosenbaum J., Hornig-Rohan M. (1996). TRH stimulation test as a predictor of acute and long-term antidepressant response in major depression. J. Affect. Disord..

[4] Shelton R.C. (1999). Treatment options for refractory depression. J. Clin. Psychiatry.

[5] Birkeland J.A., Swift F., Tovsrud N., Enger U., Lunde P.K., Qvigstad E., Levy F.O., Sejersted O.M., Sjaastad I. (2007). Serotonin increases L-type Ca^2+^ current and SR Ca^2+^ content through 5-HT_4_ receptors in failing rat ventricular cardiomyocytes. Am. J. Physiol. Heart Circ. Physiol..

[6] Hibicke M., Landry A.N., Kramer H.M., Talman Z.K., Nichols C.D. (2020). Psychedelics, but Not Ketamine, Produce Persistent Antidepressant-Like Effects in a Rodent Experimental System for the Study of Depression. ACS Chem. Neurosci..

[7] Carhart-Harris R.L., Bolstridge M., Rucker J., Day C.M., Erritzoe D., Kaelen M., Bloomfield M., Rickard J.A., Forbes B., Feilding A. (2016). Psilocybin with psychological support for treatment-resistant depression: An open-label feasibility study. Lancet Psychiatry.

[8] Ross S., Bossis A., Guss J., Agin-Liebes G., Malone T., Cohen B., Mennenga S.E., Belser A., Kalliontzi K., Babb J. (2016). Rapid and sustained symptom reduction following psilocybin treatment for anxiety and depression in patients with life-threatening cancer: A randomized controlled trial. J. Psychopharmacol..

[9] Reiche S., Hermle L., Gutwinski S., Jungaberle H., Gasser P., Majić T. (2018). Serotonergic hallucinogens in the treatment of anxiety and depression in patients suffering from a life-threatening disease: A systematic review. Prog. Neuropsychopharmacol. Biol. Psychiatry.

[10] Carhart-Harris R.L., Roseman L., Bolstridge M., Demetriou L., Pannekoek J.N., Wall M.B., Tanner M., Kaelen M., McGonigle J., Murphy K. (2017). Psilocybin for treatment-resistant depression: fMRI-measured brain mechanisms. Sci. Rep..

[11] Van Court R.C., Wiseman M.S., Meyer K.W., Ballhorn D.J., Amses K.R., Slot J.C., Dentinger B.T.M., Garibay-Orijel R., Uehling J.K. (2022). Diversity, biology, and history of psilocybin-containing fungi: Suggestions for research and technological development. Fungal Biol..

[12] Brown R.T., Nicholas C.R., Cozzi N.V., Gassman M.C., Cooper K.M., Muller D., Thomas C.D., Hetzel S.J., Henriquez K.M., Ribaudo A.S. (2017). Pharmacokinetics of escalating doses of oral psilocybin in healthy adults. Clin. Pharmacokinet..

[13] Garcia-Romeu A., Barrett F.S., Carbonaro T.M., Johnson M.W., Griffiths R.R. (2021). Optimal dosing for psilocybin pharmacotherapy: Considering weight-adjusted and fixed dosing approaches. J. Psychopharmacol..

[14] Passie T., Seifert J., Schneider U., Emrich H.M. (2002). The pharmacology of psilocybin. Addict. Biol..

[15] Halberstadt A.L., Geyer M.A. (2011). Multiple receptors contribute to the behavioral effects of indoleamine hallucinogens. Neuropharmacology.

[16] Neumann J., Dimov K., Azatsian K., Hofmann B., Gergs U. (2024). Effects of psilocin and psilocybin on human 5-HT_4_ serotonin receptors in atrial preparations of transgenic mice and humans. Toxicol. Lett..

[17] van Amsterdam J., Opperhuizen A., van den Brink W. (2011). Harm potential of magic mushroom use: A review. Regul. Toxicol. Pharmacol..

[18] Isbell H. (1959). Comparison of the reactions induced by psilocybin and LSD-25 in man. Psychopharmacologia.

[19] Hollister L.E., Prusmack J.J., Paulsen A., Rosenquist N. (1960). Comparison of three psychotropic drugs (psilocybin, JB-329, and IT-290) in volunteer subjects. J. Nerv. Ment. Dis..

[20] Malitz S., Esecover H., Wilkens B., Hoch P.H. (1960). Some observations on psilocybin, a new hallucinogen, in volunteer subjects. Compr. Psychiatry.

[21] Rinkel M., Atwell C.R., Dimascio A., Brown J. (1960). Experimental psychiatry V. Psilocybine, a new psychotogenic drug. N. Engl. J. Med..

[22] Rouzaud-Laborde C., Delmas C., Pizzinat N., Tortosa F., Garcia C., Mialet-Perez J., Payrastre B., Sié P., Spreux-Varoquaux O., Sallerin B. (2015). Platelet activation and arterial peripheral serotonin turnover in cardiac remodeling associated to aortic stenosis. Am. J. Hematol..

[23] Mialet-Perez J., Bianchi P., Kunduzova O., Parini A. (2007). New insights on receptor-dependent and monoamine oxidase-dependent effects of serotonin in the heart. J. Neural Transm..

[24] Weninger S., De Maeyer J.H., Lefebvre R.A. (2013). Influence of phosphodiesterases and cGMP on cAMP generation and phosphorylation of phospholamban and troponin I by 5-HT_4_ receptor activation in porcine left atrium. Naunyn Schmiedebergs Arch. Pharmacol..

[25] Weninger S., Van Craenenbroeck K., Cameron R.T., Vandeput F., Movsesian M.A., Baillie G.S., Lefebvre R.A. (2014). Phosphodiesterase 4 interacts with the 5-HT_4(b)_ receptor to regulate cAMP signaling. Cell Signal..

[26] Kaumann A.J., Lynham J.A., Brown A.M. (1995). Labelling with [125I]-SB 207710 of a small 5-HT_4_ receptor population in piglet right atrium: Functional relevance. Br. J. Pharmacol..

[27] Bom A.H., Duncker D.J., Saxena P.R., Verdouw P.D. (1988). 5-Hydroxytryptamine-induced tachycardia in the pig: Possible involvement of a new type of 5-hydroxytryptamine receptor. Br. J. Pharmacol..

[28] Nef H.M., Möllmann H., Troidl C., Kostin S., Voss S., Hilpert P., Behrens C.B., Rolf A., Rixe J., Weber M. (2009). Abnormalities in intracellular Ca^2+^ regulation contribute to the pathomechanism of Tako-Tsubo cardiomyopathy. Eur. Heart J..

[29] Li S., Ma Q.B., Tian C., Ge H.X., Liang Y., Guo Z.G., Zhang C.D., Yao B., Geng J.N., Riley F. (2019). Cardiac arrhythmias and cardiac arrest related to mushroom poisoning: A case report. World J. Clin. Cases.

[30] Ayme-Dietrich E., Marzak H., Lawson R., Mokni W., Wendling O., Combe R., Becker J., El Fertak L., Champy M.-F., Matz R. (2015). Contribution of serotonin to cardiac remodeling associated with hypertensive diastolic ventricular dysfunction in rats. J. Hypertens..

[31] Yabanoglu S., Akkiki M., Seguelas M.H., Mialet-Perez J., Parini A., Pizzinat N. (2009). Platelet-derived serotonin drives the activation of rat cardiac fibroblasts by 5-HT_2A_ receptors. J. Mol. Cell. Cardiol..

[32] Jaffré F., Callebert J., Sarre A., Etienne N., Nebigil C.G., Launay J.M., Maroteaux L., Monassier L. (2004). Involvement of the serotonin 5-HT_2B_ receptor in cardiac hypertrophy linked to sympathetic stimulation. Circulation.

[33] Qvigstad E., Brattelid T., Sjaastad I., Andressen K.W., Krobert K.A., Birkeland J.A., Sejersted O.M., Kaumann A.J., Skomedal T., Osnes J.B. (2005). Appearance of a ventricular 5-HT_4_ receptor-mediated inotropic response to serotonin in heart failure. Cardiovasc. Res..

[34] Banskota S., Ghia J.E., Khan W.I. (2019). Serotonin in the gut: Blessing or a curse. Biochimie.

[35] Levy F.O., Qvigstad E., Krobert K.A., Skomedal T., Osnes J.-B. (2008). Effects of serotonin in failing cardiac ventricle: Signaling mechanisms and potential therapeutic implications. Neuropharmacology.

[36] Benfey B.G., Cohen J., Kunos G., Vermes-Kunos I. (1974). Dissociation of 5-hydroxytryptamine effects on myocardial contractility and cyclic AMP accumulation. Br. J. Pharmacol..

[37] Hurtado K., Scholpa N.E., Schnellmann J.G., Schnellmann R.G. (2024). Serotonin regulation of mitochondria in kidney diseases. Pharmacol. Res..

[38] De Deurwaerdère P., Di Giovanni G. (2020). Serotonin in health and disease. Int. J. Mol. Sci..

[39] Cirillo C., Vanden Berghe P., Tack J. (2011). Role of serotonin in gastrointestinal physiology and pathology. Minerva Endocrinol..

[40] Eipel C., Abshagen K., Vollmar B. (2010). Regulation of hepatic blood flow: The hepatic arterial buffer response revisited. World J. Gastroenterol..

[41] Choi W., Namkung J., Hwang I., Kim H., Lim A., Park H.J., Lee H.W., Han K.-H., Park S., Jeong J.-S. (2018). Serotonin signals through a gut-liver axis to regulate hepatic steatosis. Nat. Commun..

[42] Mao B., Liu S., Zhu S., Wu F., Yuan W., Yan Y., Wang B. (2024). The Janus face of serotonin: Regenerative promoter and chronic liver disease aggravator. Heliyon.

[43] Saraswati S., Martínez P., Serrano R., Mejías D., Graña-Castro O., Díaz R.Á., Blasco M.A. (2024). Renal fibroblasts are involved in fibrogenic changes in kidney fibrosis associated with dysfunctional telomeres. Exp. Mol. Med..

[44] Dahmane E., Hutson P.R., Gobburu J.V.S. (2021). Exposure-response analysis to assess the concentration-QTc relationship of psilocybin/psilocin. Clin. Pharmacol. Drug Dev..

[45] Becker A.M., Holze F., Grandinetti T., Klaiber A., Toedtli V.E., Kolaczynska K.E., Duthaler U., Varghese N., Eckert A., Grünblatt E. (2022). Acute effects of psilocybin after escitalopram or placebo pretreatment in healthy subjects. Clin. Pharmacol. Ther..

[46] Yerubandi A., Thomas J.E., Bhuiya N.M.M.A., Harrington C., Villa Zapata L., Caballero J. (2024). Acute Adverse Effects of Therapeutic Doses of Psilocybin: A Systematic Review and Meta-Analysis. JAMA Netw. Open.

[47] Flanagan T.W., Nichols C.D. (2018). Psychedelics as anti-inflammatory agents. Int. Rev. Psychiatry.

[48] Zhou S.F., Wang B., Yang L.P., Liu J.P. (2010). Structure, function, regulation and polymorphism and the clinical significance of human cytochrome P450 1A2. Drug Metab. Rev..

[49] Telles-Correia D., Barbosa A., Cortez-Pinto H., Campos C., Rocha N.B., Machado S. (2017). Psychotropic drugs and liver disease: A critical review of pharmacokinetics and liver toxicity. World J. Gastrointest. Pharmacol. Ther..

[50] Conti C.R. (2018). Obesity and weight loss. Eur. Cardiol..

[51] Paravati S., Rosani A., Warrington S.J. (2024). Physiology, catecholamines. StatPearls.

[52] Austin E., Myron H.S., Summerbell R.K., Mackenzie C.A. (2019). Acute renal failure by confirmed Psilocybe cubensis mushroom ingestion. Med. Mycol. Case Rep..

[53] Bokhari S.R.A., Abdullah A., Zamir Z.A., Rosen R.M. (2022). A rare case of magic mushroom (psilocybin) related AKI and hypertensive emergency. J. Am. Soc. Nephrol..

[54] Szabo A. (2015). Psychedelics and immunomodulation: Novel approaches and therapeutic opportunities. Front. Immunol..

[55] Flanagan T.W., Sebastian M.N., Battaglia D.M., Foster T.P., Maillet E.L., Nichols C.D. (2019). Activation of 5-HT_2_ receptors reduces inflammation in vascular tissue and cholesterol levels in high-fat diet-fed apolipoprotein E knockout mice. Sci. Rep..

[56] Nau F., Yu B., Martin D., Nichols C.D. (2013). Serotonin 5-HT_2A_ receptor activation blocks TNF-α mediated inflammation in vivo. PLoS ONE.

[57] Nau F., Miller J., Saravia J., Ahlert T., Yu B., Happel K.I., Cormier S.A., Nichols C.D. (2015). Serotonin 5-HT_2_ receptor activation prevents allergic asthma in a mouse model. Am. J. Physiol. Lung Cell. Mol. Physiol..

[58] Coppola M., Bevione F., Mondola R. (2022). Psilocybin for treating psychiatric disorders: A psychonaut legend or a promising therapeutic perspective?. J. Xenobiot..

[59] Burmester D.R., Madsen M.K., Szabo A., Aripaka S.S., Stenbæk D.S., Frokjaer V.G., Elfving B., Mikkelsen J.D., Knudsen G.M., Fisher P.M. (2022). Subacute effects of a single dose of psilocybin on biomarkers of inflammation in healthy humans. Compr. Psychoneuroendocrinol..

[60] Inserra A., De Gregorio D., Gobbi G. (2021). Psychedelics in psychiatry: Neuroplastic, immunomodulatory, and neurotransmitter mechanisms. Pharmacol. Rev..

[61] Van De Kar L.D., Javed A., Zhang Y., Serres F., Raap D.K., Gray T.S. (2001). 5-HT_2A_ receptors stimulate ACTH, corticosterone, oxytocin, renin, and prolactin release. J. Neurosci..

[62] Hasler F., Grimberg U., Benz M.A., Huber T., Vollenweider F.X. (2004). Acute psychological and physiological effects of psilocybin in healthy humans: A double-blind, placebo-controlled dose-effect study. Psychopharmacology.

[63] Mahdanian A.A., Rej S., Bacon S.L., Ozdin D., Lavoie K.L., Looper K. (2014). Serotonergic antidepressants and perioperative bleeding risk: A systematic review. Expert Opin. Drug Saf..

[64] McCloskey D.J., Postolache T.T., Vittone B.J., Nghiem K.L., Monsale J.L., Wesley R.A., Rick M.E. (2008). Selective serotonin reuptake inhibitors: Measurement of effect on platelet function. Transl. Res..

[65] Mercado C.P., Kilic F. (2010). Molecular mechanisms of SERT in platelets: Regulation of plasma serotonin levels. Mol. Interv..

[66] Adebo M., Bonnet M., Laouej O., Defaix C., McGowan J.C., Butlen-Ducuing F., David D.J., Poupon E., Tritschler L., Gardier A.M. (2025). Psilocybin as transformative fast-acting antidepressant: Pharmacological properties and molecular mechanisms. Fundam. Clin. Pharmacol..

[67] Stahl S.M. (2013). Stahl’s Essential Psychopharmacology: Neuroscientific Basis and Practical Applications.

[68] Shelton R.C. (2006). The nature of the discontinuation syndrome associated with antidepressant drugs. J. Clin. Psychiatry.

