# Supplementary material for: Psilocibin: Current Evidence, Safety Signals, and Challenges in Assessing Potential Multi-Organ Effects
Source: Biomedicines. 2026 Jul 6;14(7):1516. doi: 10.3390/biomedicines14071516 (PMC13404590; doi:10.3390/biomedicines14071516)
Supplement: Supplementary file 1 [file biomedicines-14-01516-s001.zip › biomedicines-4277340-supplementary.pdf]

Table S1. Summary of the available evidence regarding organ-specific toxicity associated with psilocybin. The table presents the proposed biological mechanisms, reported clinical manifestations, and the type of evidence supporting each association across major organ systems. Current evidence indicates that transient cardiovascular effects are the most consistently documented adverse events, whereas evidence for hepatic, renal, immunological, hematological, and overall systemic toxicity remains limited and is derived primarily from preclinical studies, case reports, and mechanistic investigations.

Organ system	Proposed mechanism	Clinical manifestations	Supporting evidence
Cardiovascular system	5-HT2A-mediated sympathetic activation; possible electrophysiological effects	Tachycardia, hypertension, QT prolongation, rare arrhythmias	Clinical studies, animal studies, case reports
Liver	Altered metabolism and delayed clearance in hepatic impairment	Prolonged exposure, potentially enhanced adverse reactions	Experimental studies, clinical observations
Kidneys	Indirect injury secondary to dehydration, hyperthermia, sympathetic activation	AKI, hematuria, proteinuria, elevated creatinine	Case reports
Immune system	5-HT2A-mediated cytokine modulation and HPA-axis activation	Changes in inflammatory markers, increased cortisol	In vitro studies, animal studies,
Platelets/Hemostasis	Serotonergic regulation of platelet aggregation	Theoretical bleeding risk	Indirect evidence, mechanistic studies
Overall toxicity	Multiple receptor-mediated mechanisms; confounding factors	Serious organ toxicity rare	Clinical trials, observational studies, case reports
